# Oral Delivery of the Sj23LHD-GST Antigen by *Salmonella typhimurium* Type III Secretion System Protects against *Schistosoma japonicum* Infection in Mice

**DOI:** 10.1371/journal.pntd.0001313

**Published:** 2011-09-06

**Authors:** Guo Chen, Yang Dai, Jianxiang Chen, Xiaoting Wang, Bo Tang, Yinchang Zhu, Zichun Hua

**Affiliations:** 1 The State Key Laboratory of Pharmaceutical Biotechnology and School of Stomatology, Affiliated Stomatological Hospital, Nanjing University, Nanjing, People's Republic of China; 2 Key Laboratory on Technology for Parasitic Diseases Prevention and Control, Ministry of Health and Jiangsu Provincial Key Laboratory on Molecular Biology of Parasites, Jiangsu Institute of Parasitic Diseases, Wuxi, Jiangsu, People's Republic of China; 3 Changzhou High-Tech Research Institute of Nanjing University and Changzhou TargetPharma Laboratories Inc., Changzhou, People's Republic of China; McGill University, Canada

## Abstract

**Background:**

*Schistosomiasis japonica* is a zoonotic parasitic disease and oral vaccine delivery system would be benefit for prevention of this disease. Although attenuated salmonella has been used as an antigen expression vector for oral vaccine development, the membrane-bound vacuoles in which bacteria reside hinders the presentation of expressed heterologous antigens to the major histocompatibility complex (MHC) molecules. The present work used an attenuated *Salmonella typhimurium* strain VNP20009 to secretory expression of Sj23LHDGST bivalent antigen from *Schistosoma japonicum* and tested the protective efficacy against *S. japonicum* infection in orally immunized mice.

**Methodology/Principal Findings:**

Promoters (nirB or pagC) were used to express the antigen (Sj23LHDGST) and the *Salmonella* type III or α-hemolysin secretion system was employed to secrete it. The immunoblotting analysis and fluorescent microscopy revealed that the antigen was effectively expressed and delivered to the cytosol of macrophages in vitro. Among recombinant vaccine strains, an engineered VNP20009 which expressed the antigen by nirB promoter and secreted it through type III secretion system (nirB-sopE_1–104_-Sj23LHD-GST) efficiently protected against *S. japonicum* infection in a mouse model. This strain elicited a predominantly IgG_2a_ antibody response and a markedly increase in the production of IL-12 and IFN-γ. The flow cytometric analysis demonstrated that this strain caused T cell activation as evidenced by significantly increased expression of CD44 and CD69.

**Conclusion/Significance:**

Oral delivery of antigen by nirB-driven *Salmonella typhimurium* type III secretion system is a novel, safe, inexpensive, efficient and convenient approach for schistosome vaccine development.

## Author Summary

*Schistosomiasis japonica* is a zoonotic parasitic disease and occurs predominantly in Southeast Asia and China. Using a simple, cheap, yet efficient oral method to deliver the vaccine antigen would benefit to control its transmission in that the oral vaccine could be made into a preparation and mixed with feedstuffs of livestock hosts. In this study, we used an attenuated *S. typhimurium* strain VNP20009, whose safety has been demonstrated in phase I clinical trial, to express the bivalent *Schistosoma japonicum* antigen Sj23LHD-GST by an intracellular activated promoter (nirB) and deliver it to host cells through type III secretion system. After oral vaccination of this recombinant strain, efficient protection against *S. japonicum* challenge was induced in mice. Mean while, granuloma formation in the liver was improved significantly in the immunized mice. This protective immune response was Th1 specific type as evidenced by increase in the production of IL-12 and IFN-γ. This work provides an alternative *S. japonicum* vaccine for livestock and humans.

## Introduction

Schistosomiasis, a disease cause by intravascular trematodes from the schistosome species, is one of the world's major public health problems [1]. It is estimated that 200 million people in seventy countries or regions from Africa, Asia and South America are infected with schistosomes [2]. Five schistosome species infect humans including *Schistosoma (S.) japonicum*, *S. mansoni*, *S. mekongi*, *S. intercalatum*, and *S. haematobium*. Despite that numerous strategies have been devised and chemotherapeutic drugs such as praziquantel have been developed to combat this infectious disease, schistosomiasis still defies effective control [3]. It is generally agreed that chemotherapy has certain limitations and drug-resistance hampers its effectiveness [4]. Furthermore, praziquantel, the treatment of the first choice for schistosomiasis, is not useful as a preventive agent because its actions last only a few hours [3], besides, it is not effective against schistosomulum.

Several Schistosome vaccines have been developed, including DNA vaccines [5], peptide vaccines [6], recombinant protein vaccines [7] and multivalent vaccines[8]. These vaccines targeted antigens such as glutathione S-transferase (GST), triose-phosphate isomerase (SjTPI), paramyosin (Sj97), fatty acid binding protein (FABP, Sj14), and 23 kDa membrane protein (Sj23). These vaccines were tested in animal models and so far have been shown to provide only partial protection against *Schistosoma* infection with the worm reduction rates being mostly lower than 50% [3]. Therefore, there is an urgent need to develop an effective vaccine to control and prevent this parasitic disease, which exerts a heavy personal toll and an economic toll on the society.

Traditionally, oral vaccines are considered simple and inexpensive vehicles for delivering antigens to the host. Several oral vaccines have been developed for clinical use in humans including vaccines against cholera [9], polio [10] and typhoid [11]. The carriers used for antigen delivery in these vaccines include liposomes [12], attenuated *Salmonella* [13], bacterial spores [14], and biodegradable microparticles [15]. *Salmonella* (*S.*) *typhimurium* is a facultative intracellular bacterium and can become colonized in the cytosol of the host cell such as macrophage. *S. typhimurium* induces complex mucosal and systemic immune responses after oral administration and, because of this property, attenuated *S. typhimurium* strains have been used as delivery systems for heterologous antigen [16]. However, once internalized, *S. typhimurium* is confined to membrane-bound vacuoles, which hinders the presentation of expressed heterologous antigens to the major histocompatibility complex (MHC). One strategy to circumvent this restraint is the use of live attenuated *S. typhimurium* strains that translocate heterologous antigens into antigen-presenting cells by means of type III secretion system, a specialized protein secretion system that delivers a set of bacterial effector proteins into the host cell cytosol [17], [18] or *E. coli* α-hemolysin (HlyA) secretion system which is a type I secretion system and fully active in *Salmonella* [19], [20].

VNP20009 is an attenuated *S. typhimurium* strain whose safety has been demonstrated in phase I clinical trial [21]. In the present study, we used this live attenuated *S. typhimurium* strain to express chimeric proteins consisting of the secretion and translocation signals of *Salmonella* type III secreted protein *Salmonella* outer protein E (SopE) or α-hemolysin secretion protein (HlyA) fused to *S. japonicum* antigen Sj23LHD-GST, the bivalent antigen consisting of the long hydrophilic domain of Sj23 (Sj23LHD), which contains main T and B cell epitopes of Sj23 antigen [22], and GST (Sj26) in *S. japonicum*. The nitrite reductase B (nirB) and phoP activated gene C (pagC) promoter from *S. typhimurium* which are highly active in the intracellular environment of professional antigen presenting cells such as macrophage [23], were used to drive the expression of Sj23LHD-GST in *S. typhimurium*. Here, we sought to study in vitro expression of Sj23LHD-GST delivered by these recombinant *S. typhimurium* vaccine strains and characterize the immune responses elicited by Sj23LHD-GST in the immunized mice and further investigated the efficacies of the oral recombinant *S. typhimurium* vaccines against *S. japonicum* infection in a mouse model of schistosomiasis.

## Materials and Methods

### Plasmids

**Figure 1:**
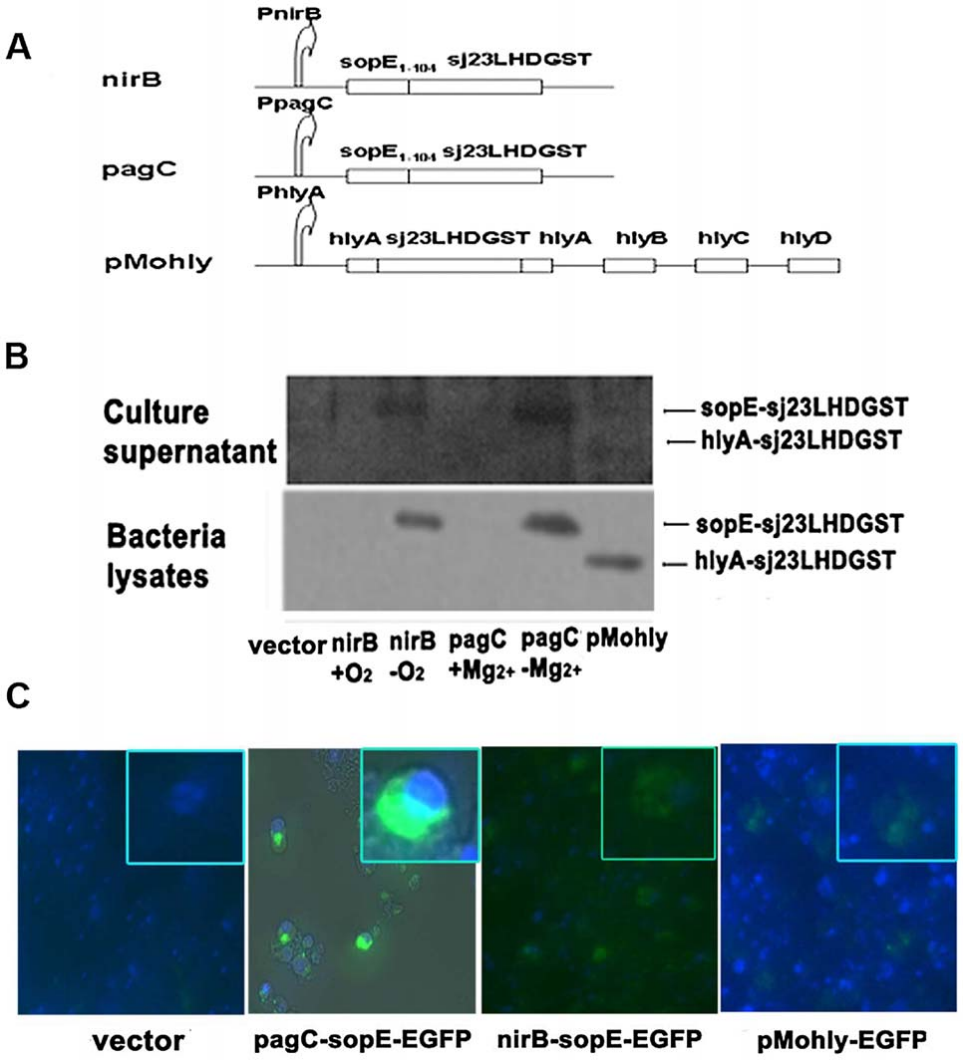
Secretory expression of recombinant Sj23LHD-GST by *S. typhimurium*. Diagram of plasmid constructs used in the study (A). nirB, pagC and pMohly represent nirB-sopE-Sj23LHD-GST, pagC-sopE-Sj23LHD-GST and pMohly1-Sj23LHD-GST constructs, respectively. Plasmids expressing the recombinant protein of Sj23LHD-GST fused to type III secretion signal of *Salmonella* outer protein E (SopE) (sopE-Sj23LHD-GST) or type I secretion signal of α-hemolysin A (HlyA) (hlyA-Sj23LHD-GST) were transformed into *Salmonella*. Whole bacteria lysates and cultured supernatants of these strains were examined for the presence of the chimeric protein sopE–Sj23LHD-GST or hlyA-Sj23LHD-GST by Western blotting as described in Methods (B). Mouse macrophage cell line RAW 264.7 was infected with *Salmonella* harboring plasmids expressing EGFP as a marker for the expressed recombinant proteins. The presence and location of EGFP in the cell were examined (C).

We cloned the nirB gene promoter (EMBL bank DQ841278.1) and the phoP-activated C (pagC) gene promoter (EMBL bank EF191162.1) from *E. coli* and *S. typhimurium* genomic DNA respectively and inserted separately into plasmid pQE30 (Qiagen) between the restriction site of XhoI and BamHI. The DNA fragment encoding the N-terminal amino acids 1–104 of sopE (sopE_1–104_) (Genebank NC003197), which are recognized as the secretion signal of SopE [17], was obtained from *S. typhimurium* genomic DNA and cloned into the restriction site between BamHI and KpnI downstream of the nirB gene promoter or pagC promoter. DNA fragment carrying the Sj23LHD-GST fusion gene (Genebank M63706, Genebank M14654), which was generated by overlap PCR, was then cloned into the restriction site between KpnI and HindIII. The newly constructed plasmids were designated plasmid nirB-sopE_1–104_-Sj23LHD-GST and pagC-sopE_1–104_-Sj23LHD-GST. Additionally, for construction of pMohly1-Sj23LHD-GST, Sj23LHD-GST was inserted into the single NsiI site of the export vector pMohly1, which was kindly provided by Prof. YX Zhang (East China University of Science and Technology). The diagrams of plasmid constructs used in the study are shown in Fig. 1A. The primers used for the above constructions are indicated in Table S1. Sj23LHD-GST gene was replaced by EGFP (Enhanced Green Fluorescent Protein) for its respective EGFP expression plasmid. Lipid A-modified (msbB^−^) auxotrophic (purI^−^) *S. typhimurium* strain VNP 20009 (ATCC, Manassas, VA, USA) was cultured in LB media. All of the plasmids and constructions were transformed into *S. typhimurium* VNP20009 by electroporation.

### Western blotting and in vitro macrophage cell line RAW264.7 infection assay

Recombinant *S. typhimurium* was grown at appropriate conditions. Proteins in the culture supernatant were prepared by precipitation with 10% trichloroacetic acid (v/v) for 1 h on ice. The bacteria lysates were prepared by sonication. Immunoblotting was performed using anti-Sj23LHDGST anti-serum as previously described [24]. Mouse anti-Sj23LHD-GST anti-serum was produced by immunizing mouse with the recombinant Sj23LHD-GST protein (rSj23LHD-GST) (produced in *E.coli*).

For macrophage infection assay, RAW264.7 cells were obtained from the American Type Culture Collection (ATCC TIB 71) and maintained in DMEM medium supplemented with 10% fetal bovine serum by routine culture methods. *S. typhimurium* harbored EGFP delivery plasmids were infected with RAW264.7 cells at a multiplicity of infection (MOI) of 100 after cells for 1 hour, then washed with phosphate buffered saline (PBS) there times to remove extracellular bacteria and further cultured with medium containing 50 µg/ml gentamicin and 10% fetal calf serum for 24 hours before observation under fluorescence microscope.

### Immunization of mice

6–8 weeks old female BALB/c mice were purchased from Shanghai Slac Laboratory Animal Co, Ltd (Shanghai, China) and maintained in specific pathogen-free, environmentally controlled conditions (22°C, a 12-h light/dark cycle with the light cycle from 6:00 to 18:00 and the dark cycle from 18:00 to 6:00) according to standard laboratory chow. Mice were randomly grouped (12 mice per group) and inoculated by gavage with 0.2 ml PBS containing 10^9^ colony forming units (CFUs) of recombinant *S. typhimurium* or *S. typhimurium* carrying pQE30 empty vector three times at an interval of 2 weeks and control mice received 0.2 ml PBS. For heterologous prime-boost immunization, two weeks after the third oral immunization with recombinant *S. typhimurium*, mice were boosted once by subcutaneous inoculation of 50 µg rSj23LHD-GST in 0.2 ml PBS. For homologous protein immunization, mice were inoculated subcutaneously with 50 µg rSj23LHD-GST three times at an interval of 2 weeks. The animals study protocol was in according with the guideline of administration of lab animals issued by the Ministry of Science and Technology (Benjing, China) and approved by the Jiangsu Institutional Animal Care and Use Committee (IACUC).

### Determination of antigen specific IgG antibody titers

Enzyme linked immunosorbent assay (ELISA) was performed to detect Sj23LHD-GST specific antibody. Briefly, rSj23LHD-GST protein was diluted in 50 mM carbonate buffer (pH 9.6) to 10 µg/ml, and 100 µl was then added to each well on 96-well plates and were incubated at 4°C overnight for antigen coating. Each plate was washed three times with PBS (pH 7.6) containing 0.05% Tween-20 (PBST), and blocked with 3% (w/v) bovine serum albumin (BSA) in PBS for 3 h at 37°C. The plates were further washed three times with PBST, and then incubated with the mouse immune sera serially diluted in PBS for detection of IgG, IgG_1_, and IgG_2a_ at 37°C for 1 h. The plates were then washed five times with PBST, followed by incubation with HRP-conjugated goat-anti-mouse IgG, IgG_1_, and IgG_2a_ (Santa Cruz Biotechnology, Santa Cruz, CA) for 45 min at 37°C. The plates were washed five times with PBST and were developed with tetramethylbenzidine substrate and read at 450 nm after the reaction was terminated using 2 M sulphuric acid.

### Determination of plasmid stability in vivo

For determination of plasmid stability in vivo, mice were orally inoculated with 1×10^9^ CFUs of non-transformed *S. typhimurium* or recombinant *S. typhimurium* strains. The spleens and Peyer's patches were collected and weighed 3, 10 and 20 d after the inoculation. The tissues were homogenized in PBS at a ratio of 5∶1 (PBS volume∶ tissue weight), and subsequently inoculated onto agar plates with or without antibiotic selection to determine the counts of recombinant and total bacteria, respectively.

### Flow cytometry assay

Mouse spleens were collected at two weeks after last immunization, then ground and sieved to obtain single cell suspension. After lysis of red blood cells, 2×10^6^ cells per well were planted in 24-well plates and cultured in RPMI 1640 containing 10% FBS. After stimulation with 20 µg/ml rSj23LHD-GST for 16 h, the cells were stained for cell surface markers using anti-CD4-PEcy5 (Phycoerythrin-Cy5), CD8-PEcy5 and CD69-FITC (fluoresceinisothiocyanate) antibodies (BD Biosciences, USA) in the dark for 45 min. After washing in PBS, the cells were resuspended and then analyzed by flow cytometry. For detection of CD44 expression, splenocytes were collected at two weeks after the last immunization and then directly stained with anti-CD44-PE (Phycoerythrin) antibodies (BD Biosciences, USA) and analyzed.

### Measurement of cytokines

For determination of IFN-γ and IL-4 concentrations, two mice were randomly selected from each group and sacrificed two weeks after the 3^rd^ immunization. Single-cell suspensions were prepared by pooling splenocytes of the two mice from each group. Red blood cells were lysed and the cells were cultured in RPMI 1640 media supplemented with 10% fetal bovine serum (FBS). A total of 6×10^5^ cells per well were cultured in triplicate wells for each group for 72 h at 37°C with 5% CO_2_ in 96-well plates in the presence of 10 µg/ml recombinant Sj23LHD protein(rSj23LHD) (produced in *E.coli*), 10 µg/ml recombinant GST protein(rGST)( produced in *E.coli*), 10 µg/ml ConA or media alone. IFN-γ and IL-4 levels in the supernatant were determined using commercially available ELISA kits (ExCell, Shanghai, China). For multiple cytokines assay using the Bio-Plex system, splenocytes from mice in each group were stimulated with rGST (10 µg/ml) for 48 h and the culture supernatants were assayed according to the manufacturer's instruction (Bio-Rad Laboratories, California, USA).

### *S. japonicum* cercariae skin penetration assays

Snails infected with *S. japonicum* were obtained from Jiangsu Institute of Parasitic Diseases and cercariae of *S. japonicum* were collected from the infected snails. Two weeks after the last immunization, every mouse in each group (12 mice per group) was challenged with 40±1 *S. japonicum* cercariae by abdominal skin penetration. Forty-two days post-challenge, all mice were sacrificed to determine worm and liver egg burdens. Adult worms were recovered by portal vein perfusion and calculated, part of the liver was cut, weighed, and digested with 5 ml 5% KOH at 37°C overnight, then dropped 50 µl liver homogenate on the glass counting slide to determine the egg number under microscope. The worm reduction rate was determined using the formula: (the average worm burden in the control group-the average worm burden in the experimental group)/the average worm burden in control group×100%. The egg reduction rate was determined using the formula: (the number of eggs per gram in the control group–the number of eggs per gram in the experimental group)/the number of eggs per gram in the control group×100%. In addition, part of the liver (1–5 cm^3^) from challenged mice was cut and fixed in 4% neutralized formaldehyde. After the tissues were embedded in paraffin, they were sectioned for hematoxylin-eosin (H&E) staining according to standard histological procedures. Granuloma formation in the livers was observed under a light microscope and at least 15 granulomas per tissue section were analyzed for granuloma area using a computerized image analysis system (Smartscape 2002, China).

### Statistics

Quantitative data were expressed as mean ± standard deviation (SD) and analyzed using the SPSS software. Paired Student's t test was performed to assess statistical significance. Differences between experimental groups were considered significant if the P value was less than 0.05.

## Results

### Recombinant *S. typhimurium* strains effectively express and deliver the antigen to the cytosol of macrophage by type III secretion system in vitro

For rational design of an effective vaccine strain, we constructed plasmid vectors expressing chimeric proteins of sopE_1–104_, N-terminal 1–104 amino acids of sopE which are recognized as the secretion signal for type III secretion system, fused to *S. japonicum* antigen Sj23LHD-GST driven by the nirB or pagC promoter (Fig. 1A). nirB is activated by anaerobic conditions and pagC is inhibited by high magnesium concentration, these two promoters are both highly active in intracellular environment of professional antigen presenting cells [23]. Additionally, we constructed the other plasmid called pMohly1-Sj23LHD-GST, which delivering the antigen through α-hemolysin (HlyA) secretion system and driven by original hlyA promoter. pMohly1 is a plasmid comprises all the components of *E. coli* α-hemolysin secretion system included hlyB, hlyC, hlyD and secretion signal of target molecule hlyA [19]. We transformed the *S. typhimurium* strain VNP20009 with the appropriate plasmid vectors and examined the efficiency of the production of the recombinant proteins by immunoblotting using anti-Sj23LHD-GST anti-serum. We found that both recombinant proteins sopE-Sj23LHD-GST and hlyA-Sj23LHD-GST driven by the respective promoter were effectively produced by *S. typhimurium* (Fig. 1B) Additionally, immunoblotting analysis of the culture supernatant revealed the presence of the chimeric protein sopE–Sj23LHDGST or hlyA-Sj23LHDGST, suggesting the efficient secretion of these chimeric proteins from the transformed bacteria.

We next examined whether the recombinant chimeric proteins were delivered to the cytosol of macrophages after infection by *S. typhimurium* harboring the appropriate plasmid vectors. We infected mouse macrophage cell line RAW 264.7 with pagC-sopE-EGFP, nirB-sopE-EGFP or pMohly1-EGFP. Florescent microscopy indicated that the recombinant proteins were effectively expressed in the infected macrophages by *S. typhimurium* carrying all three expression vectors (Fig. 1C). In addition, noticeably higher fluorescence intensity was detected by *S. typhimurium* carrying pagC-sopE-EGFP than the other two strains which indicated that this recombinant strain expressed higher protein level. These results indicate that recombinant *S. typhimurium* strains can effectively and efficiently express Sj23LHD-GST and deliver the recombinant protein to the cytosol of macrophages in vitro by type III secretion system.

### Sj23LHD-GST delivered by *S. typhimurium* driven by the nirB promoter elicits a Th1 specific humoral response in mice

**Figure 2:**
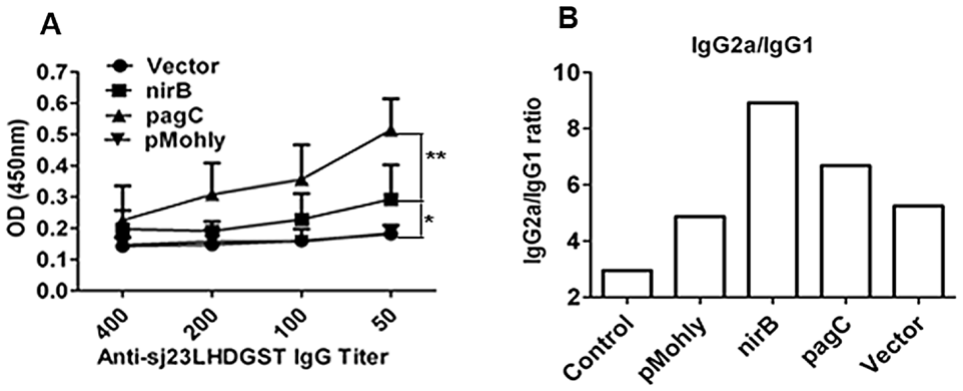
Humoral response elicited by vaccination with recombinant *Salmonella* strains. BALB/c mice were orally immunized with nirB strain, pagC strain, pMohly strain, vector strain or PBS (control) 3 times at 2 weeks interval. Two weeks after the last immunization, serum samples were collected and assayed by ELLISA. The recombinant Sj23LHDGST protein was used as the coating antigen. (A) The total antigen-specific IgG production in serial dilutions (1∶50, 1∶100, 1∶200, and 1∶400). Each bar and symbol represents the mean ± SD of sera from 8 mice. (B) The ratio of IgG2a to IgG1 in mouse serum (diluted 1∶50) from each group was calculated. The column represents the mean absorbance of IgG2a divided by the mean IgG1 absorbance of 8 animals. *P<0.05 and **P<0.01.

We further examined whether Sj23LHD-GST delivered by *S. typhimurium* could elicit effective humoral immune response in mice orally immunized with recombinant *S. typhimurium* strains. ELISA results showed that delivered antigen elicited the highest titer of serum IgG against Sj23LHD-GST in mice immunized with *S. typhimurium* harboring pagC-sopE-Sj23LHD-GST while no significant antibody response was detected in mice immunized with *S. typhimurium* harboring pMohly1-Sj23LHD-GST. In addition, Sj23LHD-GST only elicited a very moderate level of IgG in mice immunized with *S. typhimurium* harboring nirB-sopE-Sj23LHD-GST (Fig. 2A). Differently from total IgG level, the IgG_2a_∶IgG_1_ ratio (IgG_2a_/IgG_1_) in mice immunized with *S. typhimurium* harboring nirB-sopE-sj23LHD-GST was the highest (Fig. 2B). The wide dominance of IgG_2a_ isotype strongly suggests that nirB-sopE-Sj23LHD-GST strain triggered a specific Th1-type response in mice.

### The plasmid vectors expressing Sj23LHD-GST driven by the nirB promoter in *S. typhimurium* were stable in the inoculated mice

**Figure 3:**
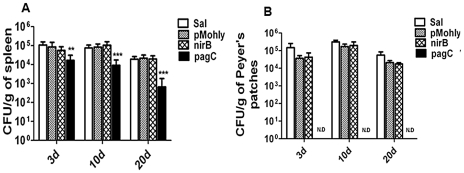
Colonization of the spleens (A) and Peyer's patches (B) of BALB/c mice following oral inoculation with recombinant *S. typhimurium* vaccines and wild type *S. typhimurium* carrying no plasmid (Sal) (white bar). The organs were collected from 3 animals 3, 10 or 20 d after inoculation and homogenized in PBS and plated onto agar for bacteria counting. Data are presented as mean ± SD, n = 3; N.D. represents no detection. **P<0.01 and ***P<0.001 when compared with non-transformed *S. typhimurium* (Sal).

Due to the fact that there was no selection pressure in the mice body, we sought to investigate whether the plasmid vectors expressing the appropriate antigens were stably present in the immunized mice by determining the colonization of recombinant *S. typhimurium* strains in murine tissues. No significant difference was found in the number of recombinant bacteria recovered from the spleens and Peyer's patches of mice inoculated with *S. typhimurium* strains harboring nirB-sopE-Sj23LHD-GST or pMohly1-Sj23LHD-GST compared with that of mice inoculated with wild type *S. typhimurium* while the number of recombinant bacteria recovered from the spleen of mice immunized with *S. typhimurium* strain harboring pagC-sopE-Sj23LHD-GST was markedly lower (Fig. 3). Furthermore, no *S. typhimurium* strain harboring pagC-sopE-Sj23LHD-GST was recovered from Peyer's patches at 3 d post inoculation (Fig. 3). The findings suggested that plasmids expressing nirB-sopE-Sj23LHD-GST were stable while those expressing pagC-sopE-Sj23LHD-GST were not stable in mice.

### Sj23LHD-GST delivered by *S. typhimurium* driven by the nirB promoter induces activation of T cells in immunized mice

**Figure 4:**
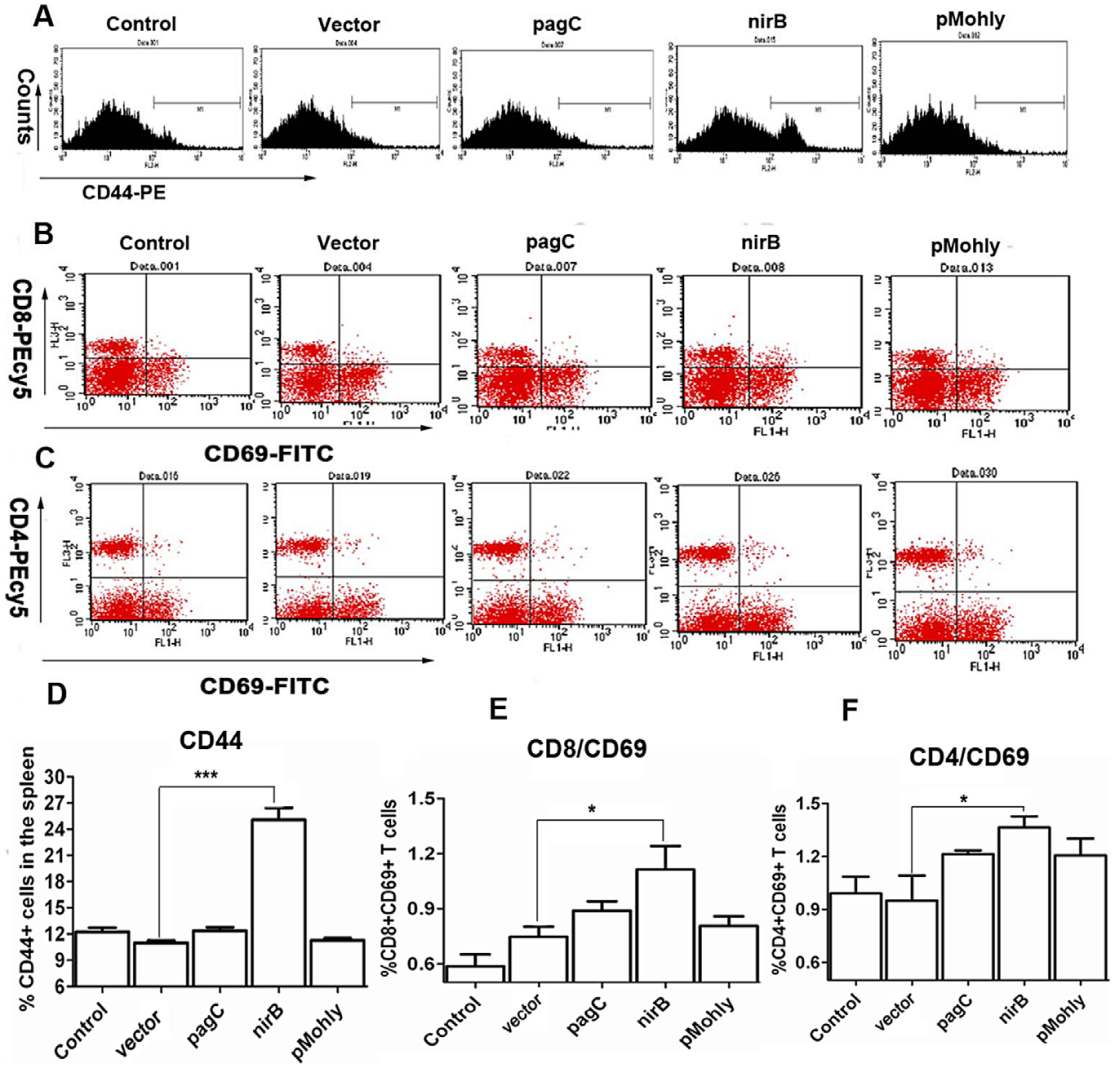
Expression of CD44 and activation of CD69. BALB/c mice were orally immunized 3 times, 2 weeks interval, with nirB strain, pagC strain, pMohly strain, vector strain or PBS (control). Splenocytes collected 2 weeks after the 3^rd^ immunization were stained with CD44-PE and assayed by flow cytometry. Histograms (A) and quantitation (D) are shown. For CD69 activation of CD4 and CD8 T cells in the presence of recombinant heterologous antigens, splenocytes collected 2 weeks after the 3^rd^ immunization were stimulated by the sj23LHDGST protein for 16 h and then doubly stained with CD4-PEcy5 and CD69-FITC or CD8-PEcy5 and CD69-FITC. Dot plots assayed by flow cytometry (B, C) and the percentage of CD8+CD69+ double positive cells (E) and CD4+CD69+ double positive cells (F) are showed. Each column represents the mean ± SD of 3 independent experiments in duplicate. *P<0.05 and ***P<0.001.

CD44 is a leukocyte homing associated adhesion molecule and a marker of activation and memory T cells [25]. We examined CD44 expression on splenocytes of mice immunized with the appropriate recombinant *S. typhimurium* strains by flow cytometry. We found a distinctly increased subpopulation of CD44^+^ cells in the splenocytes (25±2%) from mice immunized with Sj23LHD-GST delivered by *S. typhimurium* type III secretion system driven by the nirB promoter, which was significantly higher than that found in mice immunized with *S. typhimurium* strains harboring pagC-sopE-Sj23LHD-GST (12±1%) or pMohly1-Sj23LHD-GST (11±1%) or vector (11±1%) (*P*<0.01) (Fig. 4A, 4D). We further examined the expression of CD69, an early activation marker for antigen-specific stimulation of mature T cells [26], in CD4^+^ or CD8^+^ splenetic T cells following antigen stimulation from mice immunized with the appropriate recombinant *S. typhimurium* strains by flow cytometry. We observed that Sj23LHD-GST delivered by *S. typhimurium* type III secretion system driven by the nirB promoter caused a significant increase in the percentage of CD69^+^CD4^+^ (1.4±0.1%) and CD69^+^CD8^+^ (1.2±0.2%) cells compared with those of vector (0.9±0.1% for CD69^+^CD4^+^ cells and 0.5±0.1% for CD69^+^CD8^+^ cells) (*P*<0.05) (Fig. 4B–4C, 4E–4F). Though the percentage of CD69^+^CD4^+^ and CD69^+^CD8^+^ cells was higher in mice immunized with pagC-sopE-Sj23LHD-GST or pMohly1-Sj23LHD-GST, it was not statistically significantly different from that of vector.

### Sj23LHD-GST delivered by *S. typhimurium* driven by the nirB promoter protects against infection of mice by *S. japonicum*

To investigate whether our oral recombinant *S. typhimurium* vaccine strains were effective against *S. japonicum* infection, we challenged *Salmonella*-immunized mice with *S. japonicum* cercariae by abdominal skin penetration. Six weeks after the challenge, we examined the worm and egg burden in the vena mesenteria and liver of mice. The recombinant *S. typhimurium* strains harboring pagC-sopE-Sj23LHD-GST, nirB-sopE-Sj23LHD-GST or pMohly1-Sj23LHD-GST caused a 26.89%, 41.69% or 32.93% reduction, respectively, in the number of adult worms and 30.07%, 57.71% and 40.46% reduction, respectively, in the number of eggs (Table 1). Consistent with its induction of high IgG_2a_/IgG_1_ ratios, Sj23LHD-GST driven by the nirB promoter was most effective in suppressing the worm burn in the immunized mice challenged with *S. japonicum* cercariae, demonstrating the in vivo protection efficacy results from antigens delivered by *S. typhimurium* type III secretion system driven by the potent anaerobia-inducible nirB promoter.

**Table 1:** *S. japonicum* burden in mice immunized with recombinant *S. typhimurium* vaccine strains.

Groups	Mice (n)	Worms (n,% reduction)	Eggs (n, % reduction)
Control	11	30±3	122699±34214
Vector	12	26±3 (14.98)	100398±21669 (18.17)
nirB-sopE-Sj23LHD-GST	11	17±3 (41.69***)	51889±12888 (57.71***)
pagC-sopE-Sj23LHD-GST	12	22±2 (26.89*)	85805±25299 (30.07*)
pMohly1-Sj23LHD-GST	11	20±3 (32.93**)	73058±25481 (40.46**)

Data are presented as mean ± SD, n = 11–12;

*P<0.05,

**P<0.01,

***P<0.001 compared with vector.

### Immunization with *S. typhimurium*-nirB-sopE-Sj23LHD-GST induce a predominant Th1-specific cytokine response

**Figure 5:**
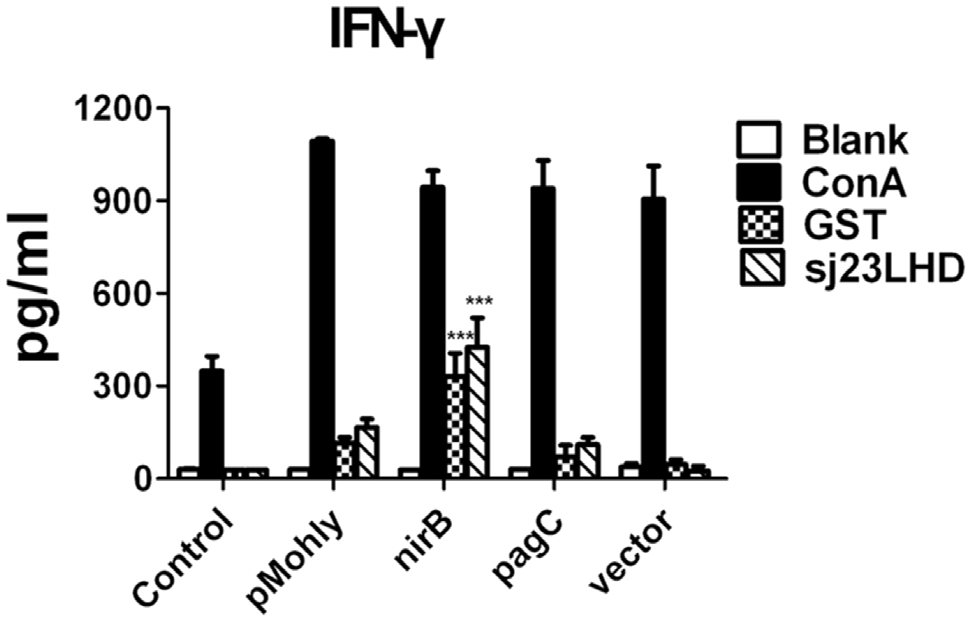
IFN-γ production by splenocytes of immunized mice. BALB/c mice were orally immunized 3 times, 2 weeks interval, with nirB strain, pagC strain, pMohly strain, vector strain or PBS (control). The splenocytes of immunized mice were collected at 2 weeks after the 3^rd^ immunization and stimulated with recombinant Sj23LHD protein (10 µg/ml), GST protein (10 µg/ml), conA (10 µg/ml) or media alone. The content of IFN-γ in the culture supernatant was measured by ELISA kit. Each column represents the mean±SD of 3 independent experiments. ***P<0.001 when compared with vector group.

To characterize the cytokine response induced by recombinant *S. typhimurium* vaccine strains, we measured the production of IFN-γ and IL-4 by splenocytes of immunized mice. We isolated and stimulated the splenocytes with recombinant Sj23LHD or GST and determined the cytokines levels by ELISA. As shown in Fig. 5, splenocytes of mice immunized with *S. typhimurium* harboring nirB-sopE-Sj23LHD-GST generated noticeably higher IFN-γ levels than splenocytes of mice immunized with the other recombinant *S. typhimurium* strains. Besides, the IL-4 production was too low to determine in the splenocyte supernatant after antigen stimulation for 72 hour (data not shown).

Many studies suggested that heterologous prime–boost vaccination by different types of vaccines containing the same antigen could be more immunogenic than traditional homologous vaccination [27]. To obtain stronger immune response to Sj23LHD-GST antigen delivered by recombinant nirB strain, we immunized mice with recombinant *S. typhimurium* harboring nirB-sopE-sj23LHD-GST followed by booster immunization with the Sj23LHD-GST protein once and examined the production of multiple cytokines by splenocytes of immunized mice by high throughput analysis. Consistent with the results of ELISA assay, we found that the splenocytes of mice immunized with *S. typhimurium*-nirB-sopE-Sj23LHD-GST or *S. typhimurium*-nirB-sopE-Sj23LHD-GST followed by booster immunization produced noticeably higher levels of Th1-specific cytokines (IL-2, IL-12 and IFN-γ) compared with those of mice immunized with the Sj23LHD-GST protein or vector (Table 2). Besides, splenocytes from mice immunized with nirB prime-protein boost regime produced higher IL-2 (121.4±6.2 pg/ml) than mice immunized with nirB strain alone (103.7±13.3 pg/ml) (Table 2).

**Table 2:** The cytokines produced by splenocytes of mice vaccinated with heterologous prime–boost regime.

Cytokines (pg/ml)	Control	Vector	Protein	nirB	nirB prime-boost	Vector prime-boost
IL-1β	175.7±56.9	277.7±34.7	210.2±2.6	220.2±35.7	235.2±45.1	161.7±34.1
IL-2	44.3±6.7	72.9±5.3	89.9±11.1	103.7±13.3*	121.4±6.2* ^,^ #	84.2±29.7
IL-3	4.1±0.5	14.1±2.8	6.9±1.3	10.1±2.3	14.4±4.6	7.5±3.1
IL-4	3.9±3.9	6.9±6.9	6.1±6.1	4.5±4.5	2.1±2.1	4.5±4.5
IL-5	2.2±0.2	2.9±0.9	5.1±1.4	2.0±1.1	1.1±0.1	1.6±0.5
IL-6	78.4±12.6	396.9±28.6	189.9±18.3	324.3±27.6	372.5±62.6	358.4±38.1
IL-9	55.7±4.1	65.2±4.3	61.0±7.8	62.8±10.1	45.0±12.6	46.1±5.5
IL-10	44.9±14.2	74.7±23.2	39.7±5.2	79.4±9.1	56.7±6.1	44.6±9.6
IL-12(p40)	39.4±2.6	51.5±6.5	34.9±5.4	74.6±8.3*	64.9±9.0*	57.8±4.6
IL-12(p70)	59.3±12.3	59.7±4.6	56.7±6.7	65.4±10.0*	72.7±4.8*	50.8±7.0
IL-13	114.9±28.6	146.9±17.2	129.8±12.4	129.5±17.1	125.2±10.6	93.0±4.5
IL-17	5.1±1.1	171.2±88.4	49.6±31.9	66.1±25.1	137.8±44.5	141.5±14.4
Eotaxin	442.6±14.0	521.4±32.5	487.2±68.2	493.2±71.2	157.6±15.7	369.7±53.0
RANTES	542.7±74.1	1488.5±218.2	670.2±26.4	1954.7±86.9	1364.7±115.9	1360.3±119.5
IFN-γ	22.2±6.3	58.2±11.8	36.7±10.2	165.0±81.6**	196.4±159.8**	24.4±9.0

Data are presented as mean ± SD, n = 3;

*P<0.05 and

**P<0.01 compared with vector;

# *P*<0.05 compared with nirB group.

### Heterologous prime–boost vaccination is more effective in protecting against infection of mice by *S. japonicum*

**Figure 6:**
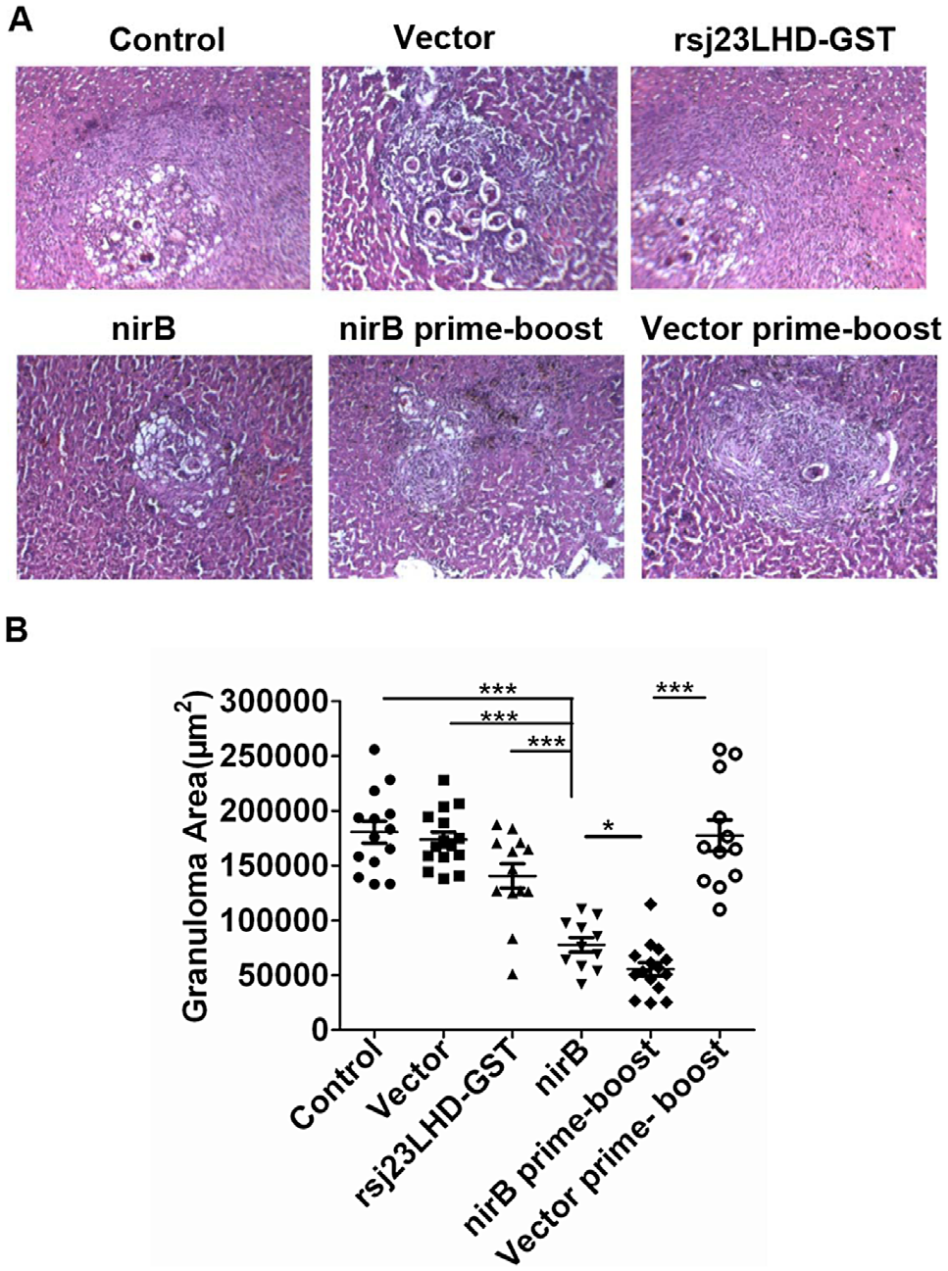
Granulomas formation in the liver. 6 weeks after *S. japonicum* challenge, the livers of mice immunized with PBS (control), vector, rSj23LHD-GST, nirB strain, nirB prime-boost or vector prime-boost were collected, fixed and sectioned for hematoxylin-eosin (H&E) staining. (A) Representative granuloma was shown (magnification, ×400) and (B) granuloma area was measured, Mean ± SD, n = 12–15. *P<0.05, and ***P<0.001.

Then, we examined the protective efficacy of nirB prime-protein boost vaccination. Analysis of the worm burden in the immunized mice showed that immunization with the Sj23LHD-GST protein and Sj23LHD-GST delivered by *S. typhimurium* type III secretion system driven by the nirB promoter caused a 21.62% and 42.73% reduction in the worm burden, respectively (Table 3) while the greatest reduction in worm burden (51.35%) was observed in mice immunized by the prime-boost method. Furthermore, the prime boost vaccination caused a 62.59% reduction in the *S. japonicum* egg burden, which was significantly higher than that by the recombinant protein alone or Sj23LHD-GST delivered by nirB driven *S. typhimurium* type III secretion system. We additionally examined granuloma formation in the liver of mice immunized with the Sj23LHD-GST protein, the recombinant *S. typhimurium* nirB strain and the heterologous prime-boost vaccination by microscopic examination of H&E-stained sections. The area of granuloma in the liver of mice immunized with nirB strain and nirB prime-boost vaccination were significantly smaller compared with those immunized with the Sj23LHD-GST protein or vector (Fig. 6). Besides, the granulomas in the liver of mice in the nirB prime-boost vaccination were the smallest (Fig. 6).

**Table 3:** *S. japonicum* burden in mice immunized with heterologous prime-boost vaccination.

Groups	Mice (n)	Worms (n, % reduction)	Eggs (n, % reduction)
Control	12	31±2	122533±22535
Vector	11	28±3 (10.37)	100797±9131 (17.74)
Protein	12	24±3 (21.62*)	83402±11364 (31.93*)
nirB	10	19±4 (42.73***)	62205±14557 (53.23***)
nirB prime-boost	11	15±3 (51.35*** ^,^ #)	45842±8810 (62.59*** ^,^ #)
Vector prime-boost	9	26±3 (14.59)	92878±23881 (24.20)

Data are presented as mean ± SD, n = 9–12;

*P<0.05 and

***P<0.001 compared with vector;

# *P*<0.05 compared with nirB group.

## Discussion

*Schistosomiasis japonica* is a zoonotic parasitic disease and infection occurs following direct contact with the larval forms of the parasite known as cercariae, which could infect humans and other mammalian hosts including buffaloes, pigs, sheep, and dogs. The eggs in feces released from the livestock hosts provide the main mode of transmission and veterinary use should be firstly considered for schistosome vaccine design [3]. For veterinary vaccine development, oral inoculation is convenient as it could be made into a preparation and mixed with feedstuffs. Additionally, schistosome infection occurs predominantly in areas of rural poverty in sub-Saharan Africa, Southeast Asia and tropical regions of the Americas [28]. Attenuated *Salmonella* expressing and delivering heterologous antigen could offer an economical vehicle for vaccine development, which may greatly benefit underprivileged populations around the world and the production costs of bacterial culture, operation and transport are far lower than those of recombinant antigens or other types of anti-schistosome vaccines. The safety of VNP20009, an attenuated *S. typhimurium* strain used in the current study as a *S. japonicum* antigen secretory expression vector, has been confirmed in clinical cancer patients [21]. Our previous study also showed that oral administration of 10^9^ CFUs of VNP20009 caused no apparent toxicity in mice [29]. These indicate that the attenuated *S. typhimurium* strain VNP20009 could be a safe and feasible vehicle for delivery of heterologous antigens.

Attenuated *Salmonella* has been used to express and translocate heterologous antigens into antigen-presenting cells by means of bacteria secretion apparatus [18], [19]. For rational design of recombinant vaccine strains, efficiency of expression and delivery of heterologous antigens are important determinants of vaccine efficacy. The efficacy of *S. typhimurium* as an antigen delivery vehicle has been hampered by its confinement to membrane-bound vacuoles after internalization, which hinders the presentation of expressed heterologous antigens to the MHC molecules. We took advantage of *S. typhimurium* type III secretion system by fusing the target antigen to the secretion and translocation signals of *Salmonella* type III secreted protein SopE. Our fluorescent microscopy demonstrated that the bivalent Sj23LHD-GST antigen was effectively delivered to the cytosol of infected macrophages in vitro, implying that *Salmonella* type III secretion system could selectively deliver antigen to desirable subcellular compartments of the target cell, rendering possible effective antigen presentation by the MHC molecules.

One further restraint of vaccine efficacy using recombinant *S. typhimurium* strains is the potential lack of stability of the plasmid constructs in the host. In the study, we observed a more vigorous Th1-specific immune response and more potent activation of T cells elicited by Sj23LHD-GST driven by the nirB promoter. Although we observed higher levels of Sj23LHDGST production driven by the pagC promoter, only a less vigorous immune response was elicited by the construct pagC-sopE_1–104_-Sj23LHD-GST, indicating that, consistent with the most recent study [30], high-level expression of heterologous antigens does not necessarily result in optimal stimulation of immune responses and potent protection against pathogen challenge. Examination of plasmid stability in the host revealed that pagC-sopE_1–104_-Sj23LHD-GST was unstable in the internal environment of the host with an early loss of the plasmid vector at 3 days post inoculation. Therefore, sustained and stable expression of antigens by plasmid vectors is required to trigger vigorous immune response.

Though several schistosome vaccines have been developed, these vaccines so far have been shown to provide only partial protection against *Schistosoma* infection with the worm reduction rates being mostly lower than 50% [3]. Some studies have shown that ultraviolet (UV)-attenuated cercariae of *S. japonicum* produced high level protection in artiodactyls, 92% in pigs and 89.1% in cattle against *S. japonicum* infections [31], [32]. However, investigators from different laboratories have found that protection in mice induced by UV-attenuated cercariae is unstable and relatively low (25%) [33]. One recent study demonstrated that UV-attenuated cercariae of *S. japonicum* could not effectively induce a Th1 type immune response in C57BL/6 mice [34]. A main obstacle in development of a successful schistosomiasis vaccine is a lack of consensus on what type of immune response should be induced [35]. Anyway, the vaccine antigens that specifically induced Th1 immune response have been described to induce promising protection against infection in the mouse model [7], [36], [37], [38]. In our study, delivery of Sj23LHD-GST through the anaerobia-inducible nirB promoter in combination with *Salmonella* type III secretion system efficiently elicited remarkable activation of antigen specific CD4^+^ and CD8^+^ T cells and high levels IL-2, IL-12 and IFN-γ. Besides, the reduction of eotaxin was observed in mice immunized by the prime-boost vaccination and this may result from the high IFN-γ level, since IFN-γ has been reported to potently inhibit eotaxin expression [39]. This Th1 predominant immune response may underlie the in vivo efficacy of the recombinant vaccine in markedly reducing worm burden and suppressing granuloma formation in the liver of immunized mice challenged with cercariae of *S. japonicum*. We observed here a 41.69% reduction in worm burden in the mice immunized with *S. typhimurium* harboring nirB-sopE-Sj23LHD-GST. Further reduction in the worm burden was realized when mice were immunized by the heterologous prime–boost strategy with the worm burden reduced by over 50%, suggesting that the oral recombinant vaccine could be further explored for development as a possible venue for preventing Schistosomiasis.

Heterologous prime-boost vaccination strategies, which can be given with unmatched vaccine delivery system while using the same antigen, have been successfully applied to many different types of diseases, including acquired immune deficiency syndrome (AIDS) [40], tuberculosis [41] and malaria [42] and have shown greater efficacy than the homologous prime-boost approach when using the same vaccine delivery system with multi-immunization. The mechanism of the strategy has not been fully elucidated and the activation of a broad spectrum of immune responses may contribute to the enhanced efficacy. DNA vaccines are usually used for priming propose and recombinant protein vaccines or viral vaccines are applied for boosting regimes [27]. Oral vaccine used as a priming regime was rarely reported and this present study showed that heterologous prime-boost strategies using oral vaccine priming followed by boosting with recombinant protein could produce enhanced protective efficacy against *S. japonicum* infection

In summary, oral administration of recombinant *Salmonella* endowed with the capability of expressing and secreting *S. japonicum* antigen induced high protection efficacy in a mouse model of schistosome infection. Mice immunization with the recombinant *Salmonella* strain with the expression of the target antigen driven by the potent anaerobia-inducible nirB promoter and secretion of the target antigen to the cytosol of host cells through *Salmonella* type III secretion system induced vigorous Th1-specific immune responses and offered potent protection against challenge by *S. japonicum* in mice. Our findings suggest that this novel vaccine design may provide a safe, cheap, efficient and convenient approach for schistosome vaccine development.

## Supporting Information

Table S1 Sequences of the primers used in constructions.
(DOC)
