# Supplementary material for: Oral Delivery of the Sj23LHD-GST Antigen by Salmonella typhimurium Type III Secretion System Protects against Schistosoma japonicum Infection in Mice
Source: PLoS Negl Trop Dis. 2011 Sep 6;5(9):e1313. doi: 10.1371/journal.pntd.0001313 (PMC3167783; doi:10.1371/journal.pntd.0001313)
Supplement: Table S1 — Sequences of the primers used in constructions. (DOC) [file pntd.0001313.s001.doc]

**Supplementary Materials:**

Table S1. The primers used in constructions

| Primers | Sequences |
| --- | --- |
| nirB p1 | cccctcgagggttaccggcccgatcg |
| nirB p2 | cccggatccgcctcgatttcttttctattacc |
| pagC p1 | cccctcgaggttaaccactcttaataa |
| pagC p2 | cccggatccaacaactccttaatactactt |
| sopE p1 | cccggatccatgactaacataacactatc |
| sopE p2 | aaaggtacccggatctttactcgcat |
| Sj23LHDGST p1^a^ | cccggtacctacaaggataaaatcgatg |
| Sj23LHDGST p2^a^ | cccaagcttttattttggaggatggtcgc |
| Sj23LHDGST p1^b^ | ccaatgcatcgtacaaggataaaatcg |
| Sj23LHDGST p2^b^ | ccaatgcatcttttggaggatggtcgc |

a The primers used in constructions of nirB-sopE-sj23LHDGST or pagC- sopE-sj23LHDGST

b The primers used in construction of pMohly-sj23LHDGST
